# Structural and Electrocatalytic Studies of Pulsed Laser Deposited Epitaxial RuO_2_ Thin Films

**DOI:** 10.1021/acsaem.5c03420

**Published:** 2026-01-02

**Authors:** Ghanashyam Gyawali, Mengxin Liu, Ikenna Chris-Okoro, Sheilah Cherono, Wisdom Akande, Brianna Barbee, Swapnil Nalawade, Jonathan Roop, Salil Pai, Shobha Mantripragada, Veluchamy Palaniappagounder, Bishnu Prasad Bastakoti, Shyam Aravamudhan, Valentin Craciun, Maria Diana Mihai, Decebal Iancu, Dhananjay Kumar

**Affiliations:** † Department of Mechanical Engineering, 3616 North Carolina A&T State University, Greensboro, North Carolina 27411, United States; ‡ Joint School of Nanoscience and Nanoengineering, 601950 North Carolina A&T State University, Greensboro, North Carolina 27401, United States; § Department of Chemistry, North Carolina A&T State University, Greensboro, North Carolina 27411, United States; ∥ 198823 National Institute for Laser, Plasma and Radiation Physics, Romania Măgurele 060042, Magurele, Romania; ⊥ Extreme Light Infrastructure for Nuclear Physics, HH-IFIN, Magurele, Turnu Măgurele 077125, Romania; # 61789 Horia Hulubei National Institute for Physics and Nuclear Engineering, Măgurele, Măgurele, IF 077125, Romania; ¶ Faculty of Applied Sciences, National University of Science and Technology Politehnica Bucharest, 060042 Bucharest, Romania

**Keywords:** ruthenium dioxide, sapphire, pulsed laser deposition, thin films, charge-transfer

## Abstract

Two sets of high-quality epitaxial ruthenium oxide (RuO_2_) thin films with different thicknesses were synthesized in situ on cost-competitive sapphire substrates by using a pulsed laser deposition technique. The first set of films, with a thickness of 40 nm and a sheet resistance of 15.7 Ω/□, was prepared using 2100 laser pulses, while the second set of films, with a thickness of 87 nm and a sheet resistance of 6.7 Ω/□, was prepared using 4800 laser pulses. All other deposition parameters were kept the same. The post-deposition structural and morphological measurements showed that both sets of films grew at the same growth rate, had the same crystallinity, similar grain boundary density, and slightly different surface roughness. The thicker RuO_2_ films achieved an overpotential of 280 mV for the oxygen evolution reaction at a current density of 100 μA/cm^2^, comparable to or exceeding the performance of films grown on more expensive substrates. Comparatively, the thinner RuO_2_ films, which have a significantly higher charge transfer resistance (250 Ω versus 100 Ω for thick films), display a higher overpotential of 320 mV. These results indicate that the lower electrical resistance of thicker films promotes charge transfer through the film body, leading to superior electrocatalytic properties.

## Graphical abstract

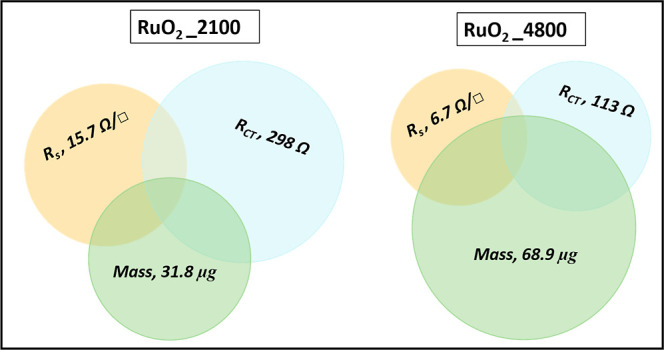

## 1 Introduction

The growing global energy demand, coupled with the environmental consequences of large fossil fuel consumption, has intensified the search for clean and sustainable energy alternatives.^1–6^ Among the emerging technologies, hydrogen production through electrochemical water splitting has garnered significant attention due to its potential to deliver a carbon-free energy carrier. Water splitting involves two half-cell reactions: the hydrogen evolution reaction (HER) and the oxygen evolution reaction (OER). Of the two half-cell reactions, the OER is kinetically sluggish, more energy-intensive, and less efficient.^7–9^ Therefore, there are widespread efforts to improve OER performance for advancing hydrogen-based energy technologies.^5,10^ Electrocatalysts in thin film form with significantly more control in crystallinity, orientation, and defects over bulk and nanomaterials offer opportunities to understand the fundamentals of HER and OER to enhance the overall performance of both reactions.^11–13^ In this study, we report the fabrication and characterization of ruthenium oxide thin films on *c*-plane sapphire substrates using a pulsed laser deposition (PLD) technique, which stands out for its precision in transferring complex target compositions onto substrates, allowing control over film thickness, surface orientation, and crystallinity. The synthesis of high-quality ruthenium oxide (RuO_2_) thin films on sapphire substrates via pulsed laser deposition (PLD) is relatively less studied and presents a technologically attractive and economically viable alternative for developing high-performance OER catalysts.^14,15^ The importance of high-quality RuO_2_ films on sapphire substrates stems from two aspects. The first aspect is associated with the cost of sapphire (single crystal alumina, Al_2_O_3_) substrates, which is almost an order of magnitude lower than other substrates commonly used for the growth of films for electrocatalysis studies, such as TiO_2_,^16–18^ MgO,^19^ and SrTiO_3_.^20^ The prices of commonly used substrates for thin film electrocatalysts are summarized in Table S1. The second aspect is associated with the importance of sapphire as technologically important substrate material. Polycrystalline silicon films on a silicon substrate, separated by a thin oxide insulator layer, are called SIS and have gained strong competition from silicon on sapphire (SOS) technology in the semiconductor industry. After silicon, there is no other substrate material than sapphire which can be produced in a single crystal wafer shape (as big as 6 in. diameter, as opposed to silicon, which is available in wafer sizes up to 18″ diameter).

The selection of RuO_2_ as a candidate material in this research arises from its capability to stabilize the OER intermediates through electroadsorption. In this process, chemical adsorption occurs simultaneously with electron transfer.^21–24^ This unique characteristic contributes to the pseudocapacitive character of the RuO_2_, making it a promising material for charge storage applications.^25,26^ However, gaining molecular-level insights into the electroadsorption mechanism on RuO_2_ films remains challenging. Specifically, well-defined experimental systems are essential to disentangle the observed redox features and attribute them to distinct electroadsorption processes.^27,28^ Our study has shown that sapphire substrates can provide a chemically stable and lattice-compatible platform that supports epitaxial growth of RuO_2_ films with desired orientation.^29^

The present study has specifically focused on the electrocatalytic performance of two sets of RuO_2_ films with identical electrical parameters in terms of the resistivity and carrier concentration. The two sets of films also have the same structural parameters in terms of crystallinity, surface orientation, composition, and surface roughness. The two sets of films differed in absolute values of their room temperature sheet resistances (15.7 Ω/□ vs 6.7 Ω/□) and thicknesses (40 and 87 nm), which were brought about by growing them using different numbers of laser pulses (2100 and 4800) and keeping all other deposition parameters the same, such as growth temperature, chamber pressure, atmosphere, and laser energy conditions. The thicknesses of both RuO_2_ films are less than the critical thickness of ∼100 nm beyond which the linear sweep voltammetry (LSV) current density at 1.81 V vs RHE decreases (Figure S1). A detailed study of the effect of RuO_2_ film thickness, in the range of 4–300 nm, on the electrochemical properties is underway in our laboratory. Each set of RuO_2_ films was produced at least three times and checked for electrical, structural, and electrocatalytic properties to establish the consistency of the results presented. The study has enabled us to gain an understanding of the role of the sample’s resistance in the charge transfer process between the electrode surface and electrolyte. These films have been found to exhibit overpotential as low as 280 mV at a current density of 100 μA/cm^2^ for OER, matching or surpassing the values reported for RuO_2_ films grown on other commonly used substrates.^30–32^

## 2 Experimental Section

### 2.1 Method, Materials, and Thin Film Deposition

RuO_2_ thin films were grown using a krypton fluoride (KrF) excimer laser (coherent complex pro, λ = 248 nm, pulse duration 25 ns) as the irradiation source on a solid target, the schematic of which is shown in Figure S2.^33,34^ In our PLD experiments,^34^ a commercially available high-purity (99.99%) RuO_2_ pellet (one inch diameter, one-quarter inch thickness) was used as the target, and 10 mm × 10 mm × 0.5 mm *c*-plane (0001-oriented) sapphire (Al_2_O_3_) pieces were used as the substrate. The substrates were ultrasonically cleaned in acetone and isopropanol, each for 20 min, followed by drying with a stream of nitrogen gas. All the depositions were conducted using previously optimized parameters, including a substrate temperature of 600 °C, a laser energy density of 2.5 J/cm^2^, a pulse repetition rate of 10 Hz, a deposition pressure of 75 mTorr of oxygen, and post-deposition cooling in 40 Torr of oxygen. Two sets of RuO_2_ films with different thicknesses were fabricated using 2100 and 4800 laser pulses while keeping all other PLD parameters constant. For brevity, these samples are referred to as RuO_2__2100 and RuO_2__4800, or thin and thick RuO_2_ films, respectively. The post-deposition thicknesses of these films were measured using X-ray reflectivity (XRR) and surface profilometry, which were found to be 40 and 87 nm for RuO_2__2100 and RuO_2__4800 samples, respectively.

### 2.2 Structural Characterization

The structural properties and film–substrate orientation were investigated by using the X-ray diffraction (XRD) technique. The XRD measurements were performed using an X-ray diffractometer (Rigaku Smartlab, Cu Kα radiation λ ≈ 1.5406 Å, applied voltage of 40 kV). The data were collected at a scan rate of 2° per minute. The unit lattice models and film–substrate epitaxial relationship were developed using visualization for electronic and structural analysis (VESTA).^35–38^ The electrical transport properties were measured by using the van der Pauw configuration and a Hall effect measurement system (ECOPIA, HMS-5300). The electrical resistivity measurements were carried out in a zero magnetic field, while Hall measurements were carried out at various temperatures (300–570 K) under a constant magnetic field of 0.565 T. The non-Rutherford backscattering spectrometry (NRBS) measurements were carried out at the 3 MV Tandetron from IFIN-HH, under high vacuum (10^–6^ mbar), using a collimated ^4^He^2+^ beam at 3.038 MeV.^39,40^ In contrast to conventional Rutherford backscattering spectrometry, at the resonance energy of the ^16^O­(α,α)^16^O nuclear reaction that was used for these measurements, the cross-section deviates strongly from Rutherford behavior, thereby improving the detection sensitivity of light elements such as oxygen in a heavier-matrix film.^41^ The α particles were detected with a passivated, ion-implanted planar silicon (PIPS) detector, placed at 165°, with respect to the incident beam direction. In order to avoid channeling effects, samples were tilted by 7° with respect to the incident beam axis. A total integrated charge of 20 μC was collected for each measurement. Typical beam currents were adjusted at ∼20 nA on target and selected to provide acceptable counting statistics while avoiding beam-induced damage or sample heating. Quantitative analysis of the backscattering spectra was carried out using the SIMNRA software.^42^

### 2.3 Electrochemical Measurements

The electrochemical properties of RuO_2_ thin films were evaluated using a three-electrode configuration employing an electrochemical workstation potentiostat (Bio-Logic, SP-300). RuO_2_ thin films served as the working electrode, with Ag/AgCl, saturated with KCl and calibrated to the H_2_ redox potential, and a Pt wire served as a reference and counter electrode, respectively. The electrochemical characterization involved recording cyclic voltammetry (CV), linear sweep voltammetry (LSV), electrochemical impedance spectroscopy (EIS), and chronoamperometry (CA) using the three-electrode setup as mentioned above, dipped in KOH solutions of 0.1, 0.5, and 1.0 M concentrations. The potential obtained using the Ag/AgCl reference electrode was converted to a reversible hydrogen electrode (RHE) according to the following Nernst equation:^43,44^ *E*_RHE_ = *E*_Ag/AgCl_ + 0.0591 × pH + *E*_Ag/AgCl°_, where *E*_RHE_ = calculated potential vs RHE, *E*_Ag/AgCl_ = measured potential vs Ag/AgCl, *E*°_Ag/AgCl_ = standard electrode potential of Ag/AgCl electrode (0.197 V at 25 °C), and pH = 13, 13.5, and 14 for 0.1, 0.5, and 1.0 M KOH, respectively. The LSVs were recorded at a scan rate of 10 mV s^–1,^ and the data were *iR* corrected using the following equation: *E*_*iR*corrected_ = *E*_(vs.RHE)_ – *i* × *R*_s_, where *i* = current density, *R*_s_ = resistance of the electrolytic solution (obtained from the EIS Nyquist plot). The overpotential (η) of electrocatalysts at a particular current density for the OER was calculated using the following equation: η_OER_ = (*E*_RHE_ −1.23) V vs RHE, where *E* signifies the *iR* corrected potential value. The reaction kinetics of the electrocatalyst were evaluated using the Tafel slope, which was determined by fitting the data to the Tafel equation: η = *a* + *b* log *j*, where η, *a*, *b*, and *j* are the overpotential, intercept, Tafel slope, and current density, respectively. Initially, cyclic voltammetry (CV) was recorded at different scan rates (20–200 mV s^–1^) in the non-faradaic region (0.0–0.2 V vs Ag/AgCl) for the OER.

## 3 Results and Discussion

### 3.1 Structural Properties

XRD analysis of RuO_2_ thin films deposited on *c*-plane sapphire (0001) substrates reveals strong (h00) oriented textured film growth of tetragonal (*a* = *b* ≠ *c*) rutile RuO_2_ phase. The results obtained are shown in Figure 1a,b. The observed (200) peak at 40.52° and (400) peak at 87.70° are sharp and well-defined; the sharpness of film peaks is comparable to that of the sapphire substrate peaks at 41.68° and 90.77°.^29,45^ The RuO_2_ film deposited using 2100 laser pulses (thickness ≈ 40 nm) was found to have lattice parameters of *a* = *b* = 0.445 nm and *c* = 0.307 nm, whereas the film deposited using 4800 laser pulses (thickness ≈ 87 nm) showed *a* = *b* = 0.444 nm and *c* = 0.307 nm. The value of the *c* lattice parameter was estimated using the 2theta position of the (211) peak, which is observed upon tilting the sample by an angle χ = 38.80° with respect to the film’s (200) orientation plane. The lattice mismatch ((*a*_s_ – *a*_f_)/*a*_s_, where *a*_s_ is the lattice parameter of the sapphire substrate and *a*_f_ is the lattice parameter of the film) of these films with the sapphire substrate (*a* ≈ 4.76 Å) is estimated to be 6.62%, which is below the critical ∼10% threshold mismatch value for maintaining coherent thin film epitaxial growth. The structural quality of RuO_2_ films is further verified by omega rocking curve (ORC) measurements; the results obtained are shown in the inset of Figure 1a. The full-width at half-maximum (fwhm) values of the (200) peak are 0.06° for both the RuO_2_ films made using 2100 and 4800 laser pulses, indicating no change in grain size. Thus, these results can be taken to suggest excellent crystallinity of RuO_2_ films on sapphire.^46^ The RuO_2_ films’ density, thickness, and surface roughness, estimated from the simulations using Rigaku software of the XRR curves (see inset of Figure 1b) with a three-layer model consisting of a RuO_2_ thin film layer, Al_2_O_3_ substrate layer, and a very thin surface contamination layer (over stoichiometric RuO_2_), are presented in Table 1.

**Figure 1:**
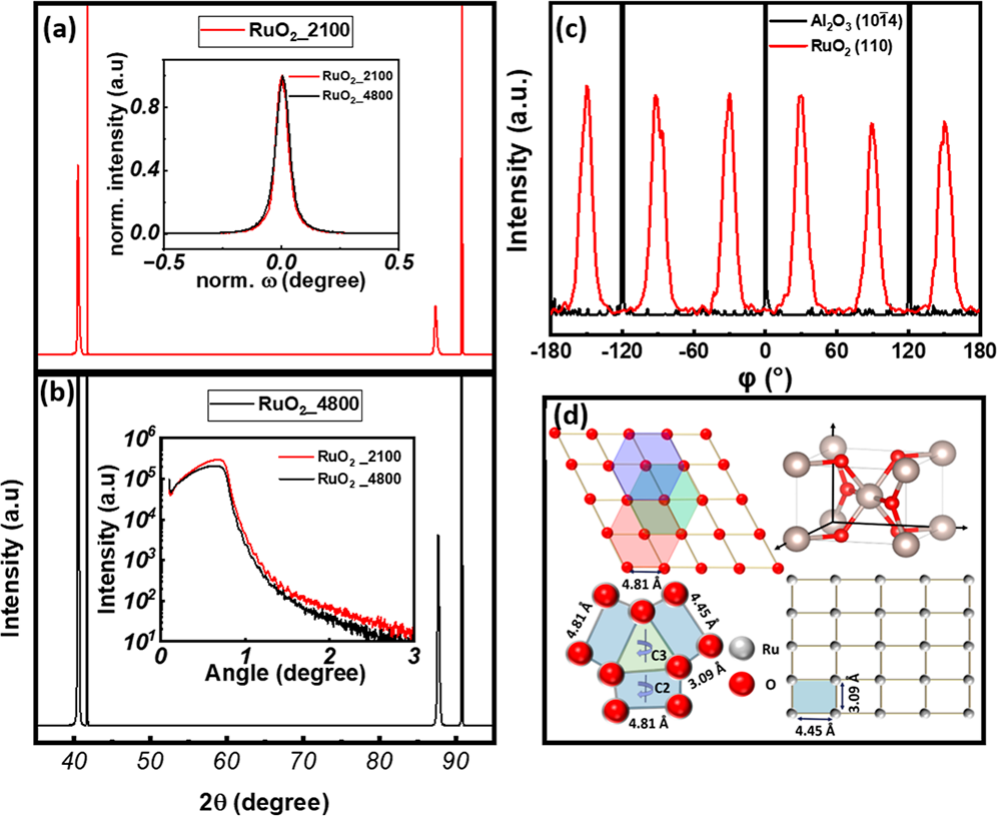
XRD patterns of (a) RuO_2__2100, (b) RuO_2__4800 samples, (c) phi-scan measurement of (110) peak of RuO_2_ thin film and (101̅4̅) peak of Al_2_O_3_ substrate, and (d) schematic representation of the epitaxial relationship between the rutile RuO_2_ film and hexagonal Al_2_O_3_ substrate. The red circles (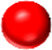) indicate oxygen atoms, and the gray circles (
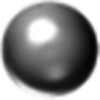
) indicate ruthenium atoms.

**Table 1:** Summary of Structural Properties of RuO_2__2100 and RuO_2__4800 Thin Films

parameters	RuO_2__2100	RuO_2__4800
thickness (nm)	40	87
fwhm (200) (°)	0.06	0.06
grain size from Scherrer formula (nm)	0.27	0.27
lattice parameter (Å)	*a* = 4.45, *c* = 3.07	*a* = 4.44, *c* = 3.07
roughness (nm)	1.96	2.88
density (g/cm^3^)	7.97	7.92

Shown in Figure 1c are the phi-scan measurements for the Al_2_O_3_ (101̅4̅) planes and RuO_2_ (110) planes. The selection of the (101̅4̅) plane for the Al_2_O_3_ substrate and (110) plane for RuO_2_ film was made due to their strong intensity and relatively smaller chi (χ) values of these planes with respect to the (0001) plane of the Al_2_O_3_ and the (100) plane of RuO_2_, respectively. The geometrical symmetry of the substrate and film planes, as well as the epitaxial relationship between the RuO_2_ film and Al_2_O_3_ substrate, are explained using their two-dimensional unit cells (Figure 1d). The appearance of three peaks from the Al_2_O_3_ substrate is attributed to the 3-fold (C3) symmetry of the triangular 2D repeat unit cells in the (0001) plane of Al_2_O_2_ (one triangle is shaded green for illustration). Six peaks corresponding to RuO_2_ are attributed to three domains of the RuO_2_ (100) plane, each having a 2-fold (C2) symmetry. As seen in Figure 1d, the smallest repeat unit cell of the (0001) plane of sapphire is a triangular plane, around which three (100) planes of RuO_2_ are aligned.^29,46^ The C3 symmetry of the (0001) plane of sapphire and the C6 symmetry of RuO_2_ (due to three domains of the (100) plane) result in 30° separations between the peaks arising from the Al_2_O_3_ substrate and RuO_2_ film, as observed in Figure 2d. It is also clear from this schematic that RuO_2_ films are under tensile stress along the *a*-axis (*a*_f_ < *a*_s_; 4.45 Å vs 4.81 Å) and compressive stress along the *c*-axis (*c*_f_ < *a*_s_; 3.09 Å vs 4.81 Å). The presence of 6 diffraction peaks in the phi scans indicates the presence of three domains with a 120° in-plane rotation to each other and rotated ±30° with respect to the (101̅4̅) sapphire substrate.

**Figure 2:**
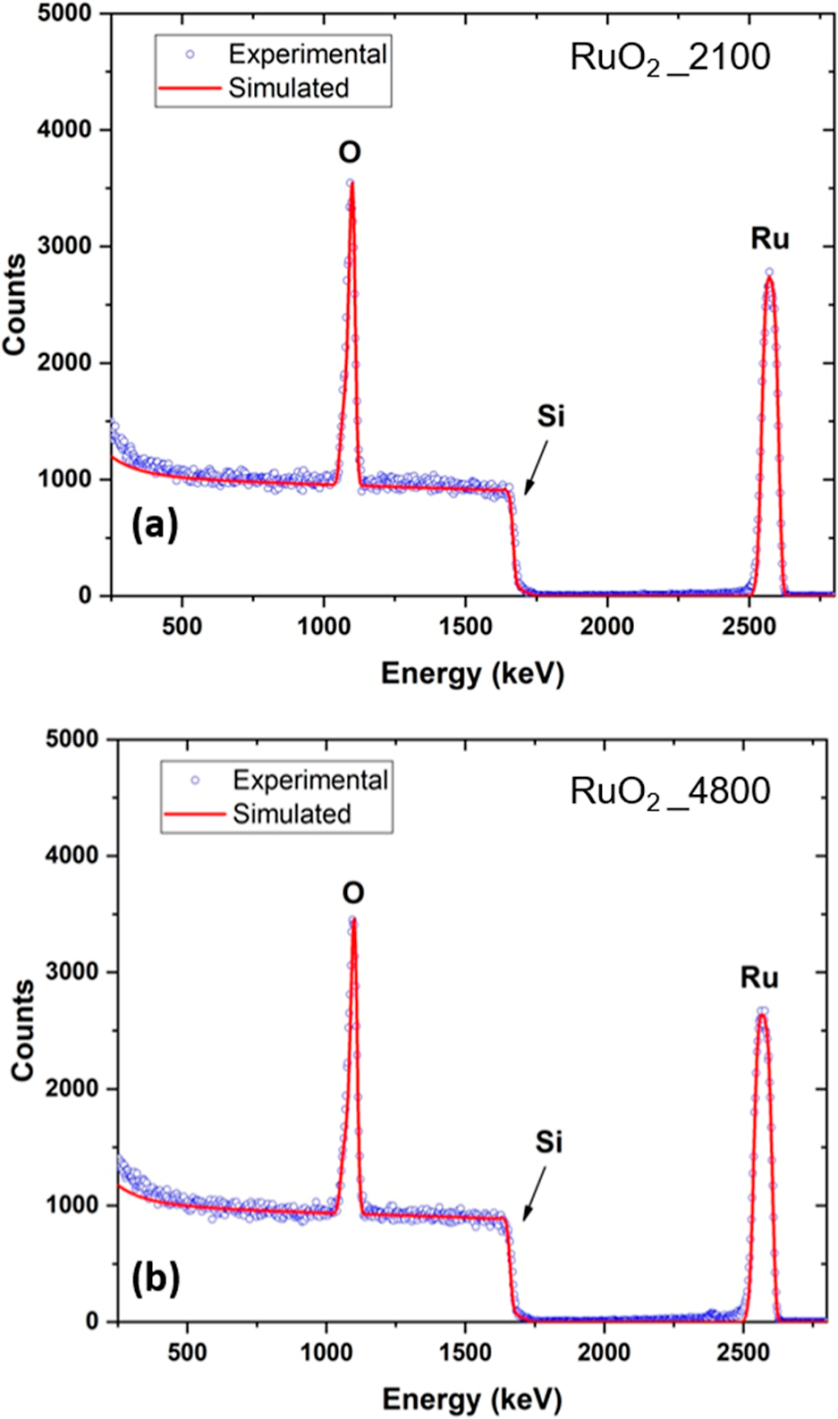
Non-Rutherford backscattering spectra acquired from (a) RuO_2__2100 and (b) RuO_2__4800 thin film samples deposited on Si substrates and their simulations (red traces).

The elemental composition and thickness of the RuO_2__2100 and RuO2_4800 samples were determined from simulated fits to the experimental NRBS spectra (Figure 2), revealing an almost identical stoichiometric film composition for both sets of films, namely, of Ru_0.34_O_0.66_ and Ru_0.33_O_0.67_, respectively. The thicknesses of the two sets of films, taking into account their densities listed in Table 1, were found to be around 44 and 85 nm for the RuO_2__2100 and RuO_2__4800 samples, respectively, which corroborate the thickness results obtained from simulations of the XRR curves recorded from these films. It should be noted that the films deposited on sapphire tend to charge during RBS, which affects the measurement; this is why we used films deposited on Si.

The structural ordering and phase purity of RuO_2__2100 and RuO_2__4800 thin film samples were further confirmed by recording their Raman spectra, which are presented in Figure 3. The Raman spectra exhibit distinct vibrational modes characteristic of the rutile phase of RuO_2_. At 512 and 623 cm^–1^, the *E*_g_ and A_1g_ modes, respectively, are observed, consistent with previously reported values for single-crystal RuO_2_.^47,48^ Among the four expected Raman-active modes, the *E*_1g_ and A_1g_ modes are clearly observed in both films, while the B_2g_ mode is below the instrument resolution in the thinner film sample (RuO_2__2100). When viewed along the *c*-axis, the spatial arrangement of ions in the (001) plane of RuO_2_ is schematically illustrated in the inset of Figure 3. The schematics highlight the symmetry of Raman-active modes at the point (zero wave vector), all of which involve only oxygen atom displacements (as shown in the inset of Figure 3). The absence of the B_1g_ mode in the thinner RuO_2_ sample may be attributed to a weak Raman scattering signal due to less material in the thinner sample. The gradual enhancement of peak intensity and the emergence of additional vibrational modes further support the improvement in structural ordering and phase purity with increasing film thickness (or deposition pulse count).

**Figure 3:**
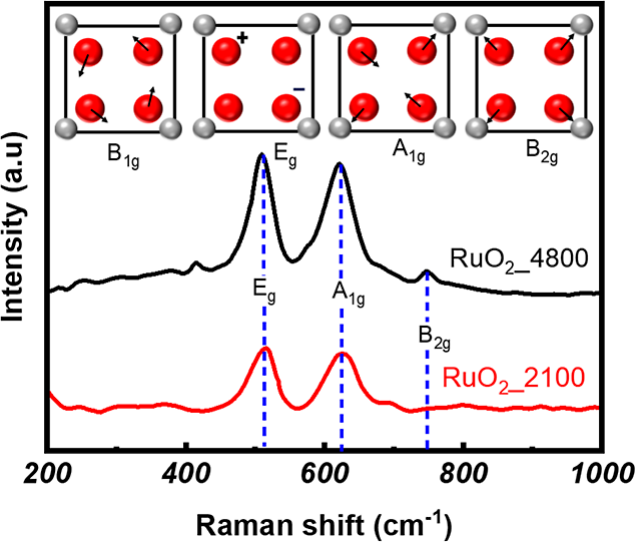
Raman spectra of RuO_2__2100 and RuO_2__4800 thin films. The inset shows the spatial arrangement of ions in the (001) plane of RuO_2_ when viewed along the *c*-axis. The red circles (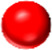) indicate oxygen atoms, and the gray circles (
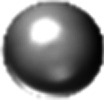
) indicate ruthenium atoms. The arrows indicate the atomic displacements associated with four Raman-active modes.

### 3.2 Electrochemical and Electrical Properties

#### 3.2.1 Cyclic Voltammetry

The cyclic voltammetry curves for the RuO_2__2100 and RuO_2__4800 samples, recorded in alkaline electrolytic media with varying concentrations, are shown in Figure 4. The voltage range (0.36–1.61 V versus RHE) selected for recording these CV curves was intended to capture all the chemical steps of a four-electron transfer reaction during water splitting, expressed in chemical scheme 1. Understanding and optimizing each of these elementary steps are vital for enhancing the efficiency of the OER and designing advanced electrocatalysts. The upper and lower limits of potential were selected to avoid chemical alteration in the RuO_2_ as reported in the literature.^2,49^ The CV curves obtained in 0.1, 0.5, and 1.0 M KOH solutions are shown in the left panel, middle panel, and right panel, respectively, of Figure 4. The first application of these CV measurements was in determining the oxygen electroadsorption energies and redox assignment of the reactions expressed in chemical scheme 1.

**Figure 4:**
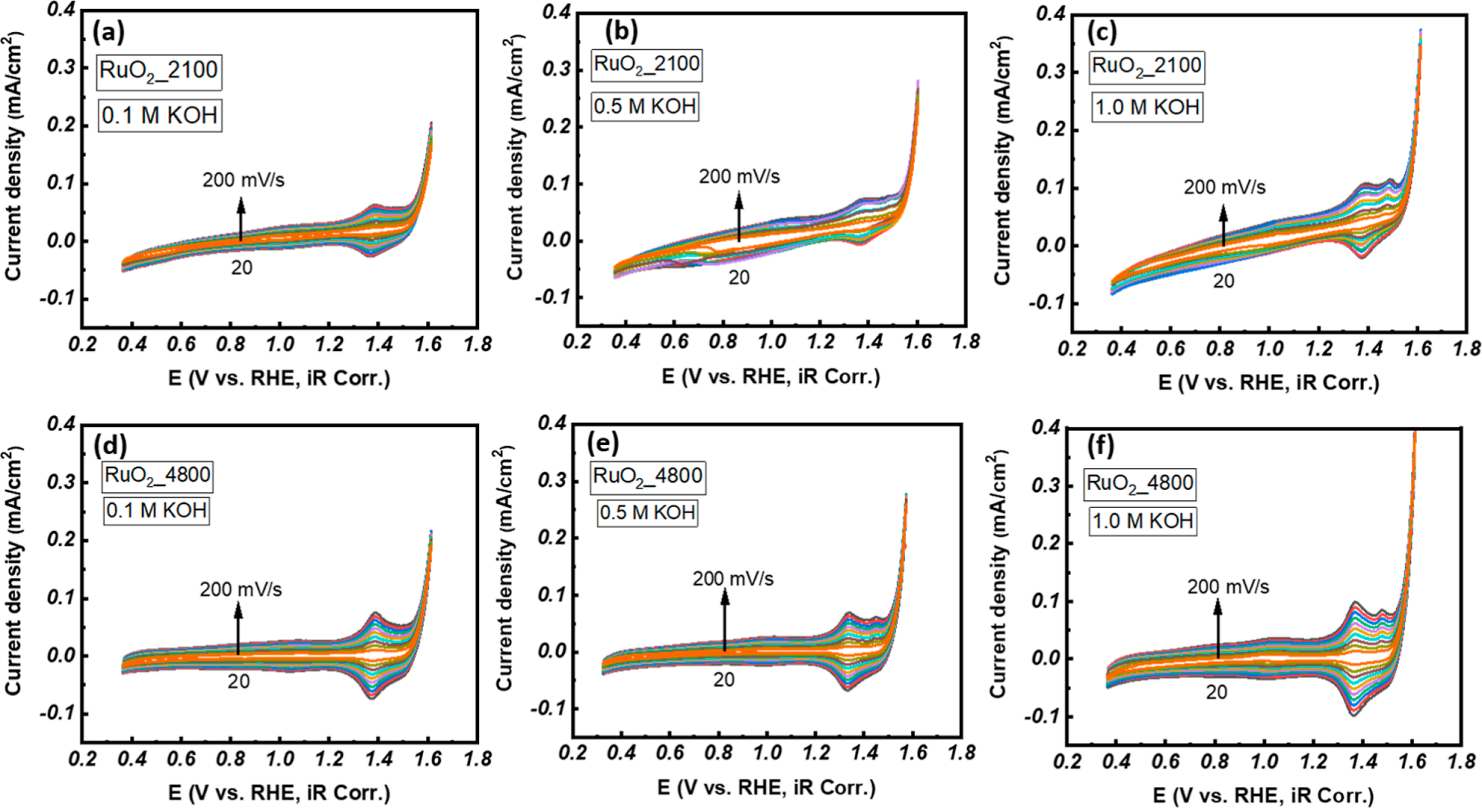
Cyclic voltammetry curves recorded at various scan rates from 20 to 200 mV s^–1^ of RuO_2__2100 in (a) 0.1 M KOH, (b) 0.5 M KOH, (c) 1.0 M KOH, and RuO_2__4800 in (d) 0.1 M KOH, (e) 0.5 M KOH, (f) 1.0 M KOH.

Chemical Scheme I:

1. OH^–^ adsorption

$${OH}^{-}+{\;}^{*}\to {OH}^{*}+e^{-}$$

2. Formation of O intermediate*

$${OH}^{*}+{OH}^{-}\to O^{*}+H_{2}O+e^{-}$$

3. OOH formation*

$$O^{*}+{OH}^{-}\to {OOH}^{*}+e^{-}$$

4. O_2_ release

$${OOH}^{*}+{OH}^{-}\to O_{2}+H_{2}O+e^{-}+{\;}^{*}$$

* represents an active site on the catalyst surface.

Initially, an OH^–^ ion adsorbs onto active site * to form an OH intermediate (OH*), releasing one electron. This is followed by the transformation of OH* to O* by bonding with OH^–^ ions in solution, concurrently producing a water molecule and releasing another electron. The third step involves the formation of an OOH* species through a nucleophilic attack by a hydroxide ion on O*, accompanied by the release of a third electron. Finally, the OOH* intermediate reacts with another OH^–^ ion to evolve molecular oxygen (O_2_), releasing the fourth electron and one more water molecule. As shown in this chemical scheme, the last step regenerates an active site, thereby promoting the first step and facilitating the continuation of the full chemical scheme.

In order to discuss the charge transfer mechanism during the OER, the CV curves were recorded at multiple scan rates ranging from 20 to 200 mV/s for both samples in all three electrolytes with different concentrations (0.1, 0.5, and 1.0 M KOH). As shown in Figure 4a–f, the current densities in the entire voltage range applied increase with an increase in the scan rate; this is true for all three electrolytic solutions. It can also be noted that the higher the concentration of the electrolytic solution, the higher the current densities. For example, the current densities of the RuO_2__2100 samples are 0.20, 0.27, and 0.38 mA/cm^2^, and the RuO_2__4800 samples are 0.22, 0.28, and 0.40 mA/cm^2^ (at 1.61 V vs RHE) in 0.1, 0.5, and 1.0 M KOH, respectively. The increase in current density is attributed to a rise in the concentration of ionic species. It seems the drag force due to surrounding ions of opposite charge is not as dominant as the effect due to an increase in ionic concentration.^50,51^

Now we return to studying the effect of voltage scan rate on the current density using Randles–Sevcik equation:^52–55^ [*i*_p_ = 0.446nFAC­(*n*FνD/RT)^0.5^ for a diffusion controlled reaction, and *i*_p_ =(*n* ^2^F^2^/4RT)­νAΓ for an adsorption-controlled reaction]. In these equations, *i*_p_ is the peak current, *n* is the number of electrons transferred in the redox process (typically 1), *A* = geometric electrode area, *D* is the diffusion coefficient, *C* is the analyte concentration, ν is the scan rate, and Γ is the surface coverage of the adsorbates. A linear dependence of the peak current density as a function of the square root or simply the scan rate can be used to distinguish between diffusion-controlled and adsorption-controlled charge transfer reaction mechanisms operating during the water splitting reactions. As shown in Figures 5a,b and S3­(a–d), current density fits well to both ν^0.5^ and ν^1^. So, a further analysis was carried out using the fitting graph of log­(*i*) versus log­(ν) to determine the charge storage mechanism:^56,57^ log­(*i*) = log­(*a*) + *b* log­(υ), where *i* is the current density, *a* is constant, *b* is slope, ν is the scan rate of applied potential. In this equation, a slope value of 0.5 indicates a diffusion-controlled process, while a slope value of 1.0 suggests a surface-controlled (capacitive) process.

**Figure 5:**
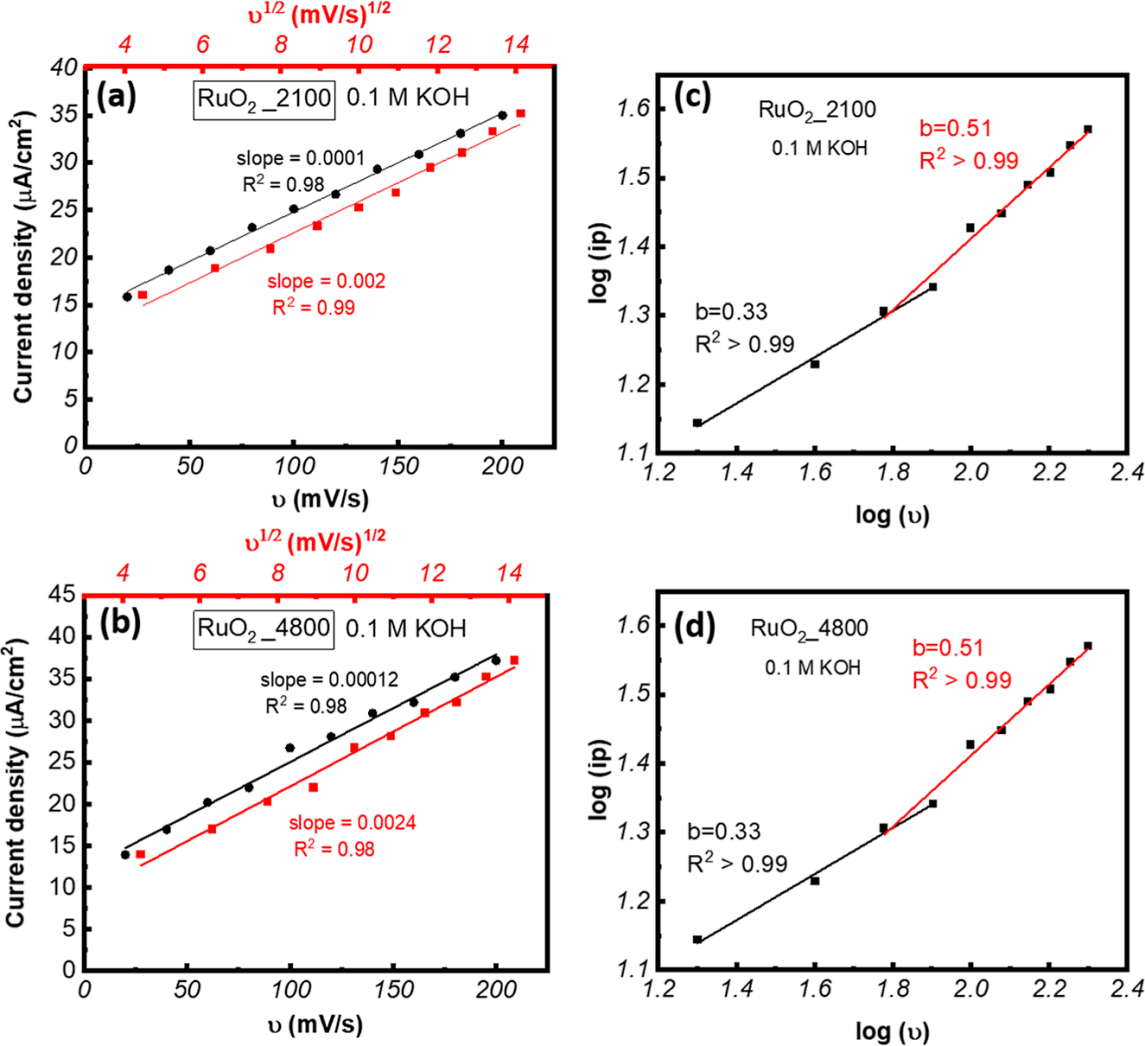
Plotting of anodic and cathodic peak current density as a function of the square root and linear of scan rates for (a) RuO_2__2100 and (b) RuO_2__4800 samples, relationship between log­(*i*) vs log­(*v*) of the (c) RuO_2__2100 and (d) RuO_2__4800 samples in 0.1 M KOH solution.

As seen in Figures 5c,d, the slope for both samples is 0.51 at higher scan rates and 0.31 at lower ones. The slope values are very similar at higher concentrations of KOH, as shown in Figure S4a–d. Thus, by plotting the experimental data of log­(*i*) vs log­(*n*) and fitting them linearly, we can quantitatively distinguish between capacitive contributions and diffusion-controlled kinetics, providing valuable insights into the electrode material’s charge-storage characteristics. The RuO_2__4800 film in 0.1 M KOH displayed the highest diffusion coefficient of 5.96 × 10^–11^ cm^2^/s, followed by the RuO_2__2100 film (3.80 × 10^–11^ cm^2^/s) in 0.1 M KOH, indicating superior ionic mobility in the case of the RuO_2__4800 sample. The CV curves were also used to determine the capacitance (*C*_DL_) of the double-layer formed between the RuO_2__2100 and RuO_2__4800 samples and various concentrated KOH electrolytes (Figure S5a–f). The calculated *C*_DL_ values (Figure S6a,b) were 62.3, 72.5, and 79.2 μF/cm^2^ for RuO_2__2100, and 68.7, 85.4, and 94.6 μF/cm^2^ for RuO_2__4800 in concentrations of KOH electrolyte of 0.1, 0.5, and 1.0 M. The capacitance was also calculated using the integrated area approach of the curve in the non-faradaic region. The average capacitance values obtained (Figure S6c,d) are 52.4, 62.1, and 73.3 μF/cm^2^ for the RuO_2__2100 sample and 57.8, 67.5, and 80.3 μF/cm^2^ for the RuO_2__4800 sample in the KOH electrolytes with concentrations of 0.1, 0.5, and 1.0 M, respectively.

#### 3.2.2 Electrochemical Impedance Spectroscopy

EIS was employed to examine the charge-transfer characteristics of RuO_2_ thin-film samples under varying applied potentials and different KOH electrolyte concentrations using Nyquist and Bode plots. Figure 6a,b present the Nyquist plots for the RuO_2__2100 and RuO_2__4800 samples, respectively. These plots show the negative imaginary impedance (-im­(*Z*)) versus the real impedance (Re­(*Z*)), along with fitting results using a standard Randles equivalent circuit.^58–61^ The simple Randles circuit that consists of solution resistance (*R*_s_), double-layer capacitance (*C*_DL_), and charge transfer resistance (*R*_CT_) is expressed as *Z* = *R*_S_ + *R*_CT_/(1 + *j*_ω_ *R*_CT_ *C*_DL_). This equation represents a system where the uncompensated resistance *R*s is in series with a parallel combination of *R*_CT_ and *C*_DL_. At high frequencies, the capacitive reactance becomes negligible and the total impedance approaches *R*_S_. At low frequencies, the capacitive impedance increases, leading to the total impedance to approach *R*_s_ + *R*_CT_. This model typically yields a semicircular Nyquist plot, with the semicircle’s diameter equal to RCT and its high-frequency intercept corresponding to *R*s, making it helpful in analyzing charge-transfer processes and interfacial properties in electrochemical systems. Notably, *R*_CT_ is directly associated with the electrocatalytic reaction kinetics of the OER, the higher and lower values of which (*R*_CT_) indicate a slower or faster reaction rate, respectively.

**Figure 6:**
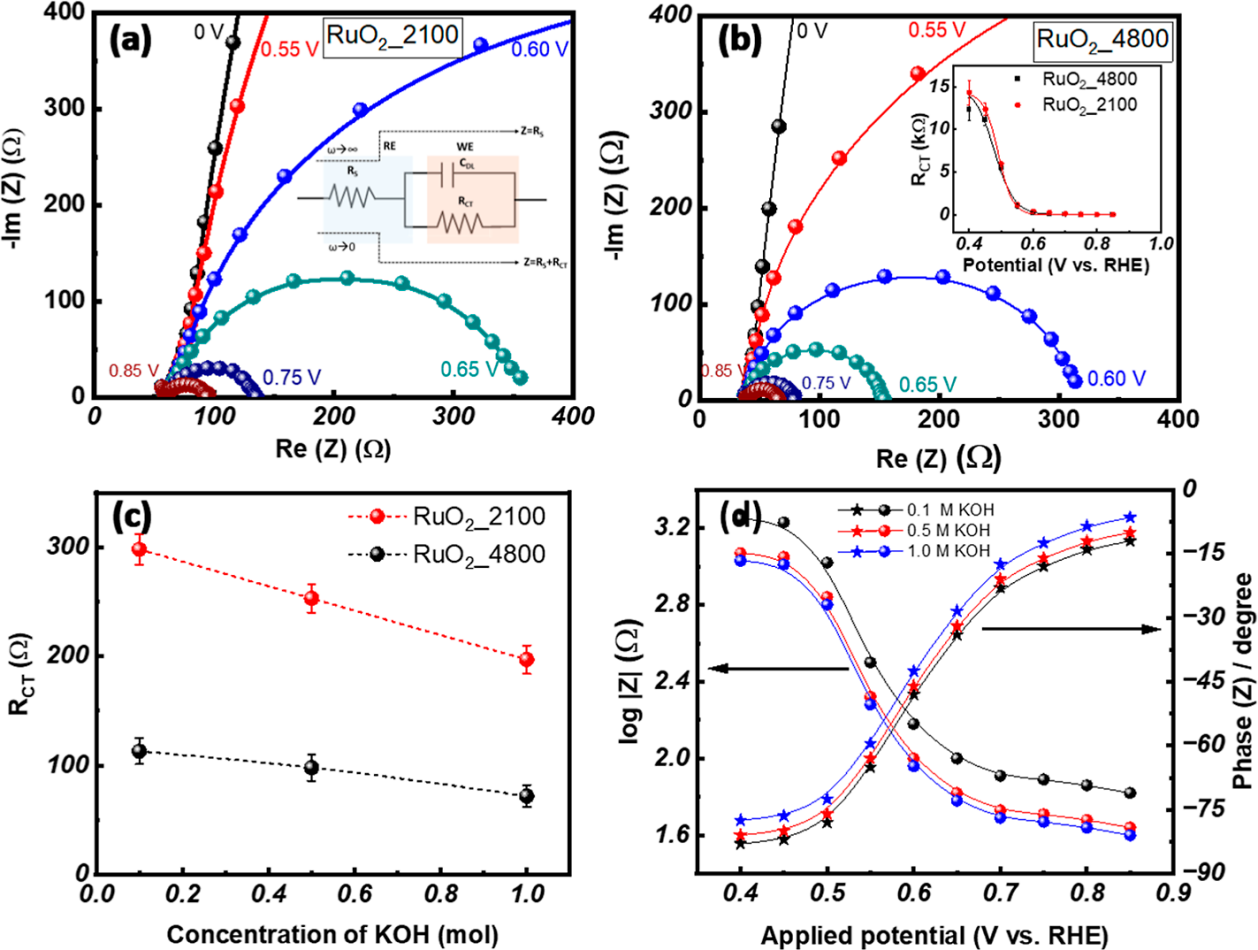
Nyquist plots under various applied potential of (a) RuO_2__2100 (inset shows representative of Randles circuit), (b) RuO_2__4800 thin films (inset shows a diagram of charge transfer resistance as a function of applied potential), (c) charge transfer resistance of RuO_2__2100 and RuO_2__2100 thin films under various KOH concentrations, and (d) impedance (|*Z*|) and phase angle as a function of applied potential for RuO_2__4800 samples in different concentrations of electrolyte.

The influence of applied potentials on the EIS response is shown in Figure 6a (RuO_2__2100 sample) and Figure 6b (RuO_2__4800 sample). At an applied potential of 0 V (i.e., open circuit), the Nyquist plot exhibits nearly linear behavior, characteristic of diffusion-limited processes, or dominant capacitive behavior at the electrode interface. As the applied potential increases, the plots begin to bend and acquire semicircular features, indicating the beginning of the Faradaic processes. This transition may reflect the requirement for a minimum activation energy for the OER to enable electron transfer at the electrode–electrolyte interface. For instance, the RuO_2__2100 sample exhibited a higher *R*_CT_ value (298 Ω) compared to the RuO_2__4800 sample (113 Ω) in 0.1 M KOH at an applied potential of 1.61 V (vs RHE). We attribute the lower charge-transfer resistance of the RuO_2__4800 sample with respect to that of the RuO_2__2100 sample to the lower absolute resistance of the former compared to that of the latter. Since a low electrical resistance of an electrode promotes rapid charge transfer and high-power density, thicker RuO_2_ films with lower electrical (ohmic) resistance facilitate faster and more efficient electron flow across the electrode–electrolyte interface. A variation in electrical resistivity between two sets of the same materials can be caused by a difference in the grain boundary density, crystallinity, and surface roughness.^62,63^ However, the difference in the resistance of thick and thin RuO2 films in the present study is believed to be due to the reductive effect of their dimensions (increase in the film thickness decrease film’s electrical resistance) and surface roughness (increase in film’s surface roughness increases film’s electrical resistance), as the two sets of films have no differences in grain size and crystallinity.

As shown in Table 1, both samples have nearly identical values for all other parameters; however, they differ by almost a factor of 2 in their four-probe electrical resistances. In the plotting as shown in Figure 6c, as the electrolyte concentration increases, the *R*_CT_ clearly decreases, which could be attributed to the enhanced ionic conductivity at higher concentrations that reduce the resistive losses and facilitate faster charge transfer kinetics, as evidenced by the reduction in the magnitude of the impedance and a shift of the maximum phase toward higher frequencies in the Bode plots.^33,64^ The Bode magnitude and phase plots, as shown in Figure S7a–c, and the plots of impedance and phase angle as a function of applied potential, as shown in Figure 6d, support our charge transfer-related arguments. The Bode phase plot shows a distinct maximum near the characteristic frequency, confirming a single time constant process typical of a Randles-type circuit. These observations highlight the critical role of electrolyte optimization and structural tuning in enhancing the electrochemical performance of RuO_2_ thin-film electrodes. The application of EIS via the Nyquist diagram has been extended to a variety of materials to elucidate the transfer kinetics of redox-probe electrons at electrode surfaces, including the enzyme-triggered formation of enzyme^60^ and MXene nanosheet-based capacitance immunoassays.^61^

#### 3.2.3 Linear Sweep Voltammetry

The electrocatalytic performance of the RuO_2_ thin films was evaluated in a N_2_-saturated KOH electrolyte with various concentrations, as presented in Figure 7. Figure 7a displays the LSV polarization curves for the OER of the RuO_2_ thin film grown at 2100 pulse numbers (RuO_2__2100). The RuO_2__2100 samples exhibited overpotentials of 340, 330, and 320 mV vs RHE under 0.1, 0.5, and 1.0 M KOH, respectively, to achieve current densities of 100 μA cm^–2^, which are significantly higher than those recorded for the RuO_2__4800 thin films (310, 290, and 280 mV vs RHE) as shown in Figure 7b, respectively. The reaction kinetics were further analyzed through Tafel slope fitting (insets Figure 7a,b). The RuO_2__2100 samples exhibited Tafel slopes of 244, 183, and 150 mV dec^–1^, which are considerably higher than the slopes (231, 144, and 115 mV dec^–1^) for RuO_2__4800 samples under 0.1, 0.5, and 1.0 M KOH, respectively. The lower Tafel slopes of RuO_2__4800 samples suggest a more favorable electron transfer process for the OER. It can be assumed that the enhanced catalytic activity of the RuO_2_ thin film is due to an increase in the laser pulse number (film thickness). This hypothesis is supported by the measured Tafel slope, which indicates that the initial electron transfer step is the rate-determining step in the OER process. Beyond a specific film thickness, the benefits of increased surface area are offset by limitations in charge transport. This phenomenon is commonly referred to as the “critical thickness” in nanoscale metal oxide electrocatalysts.^32,65,66^ At this stage, the increased electron transport path length within the bulk of the film becomes the rate-limiting factor, thereby diminishing the overall catalytic efficiency.

**Figure 7:**
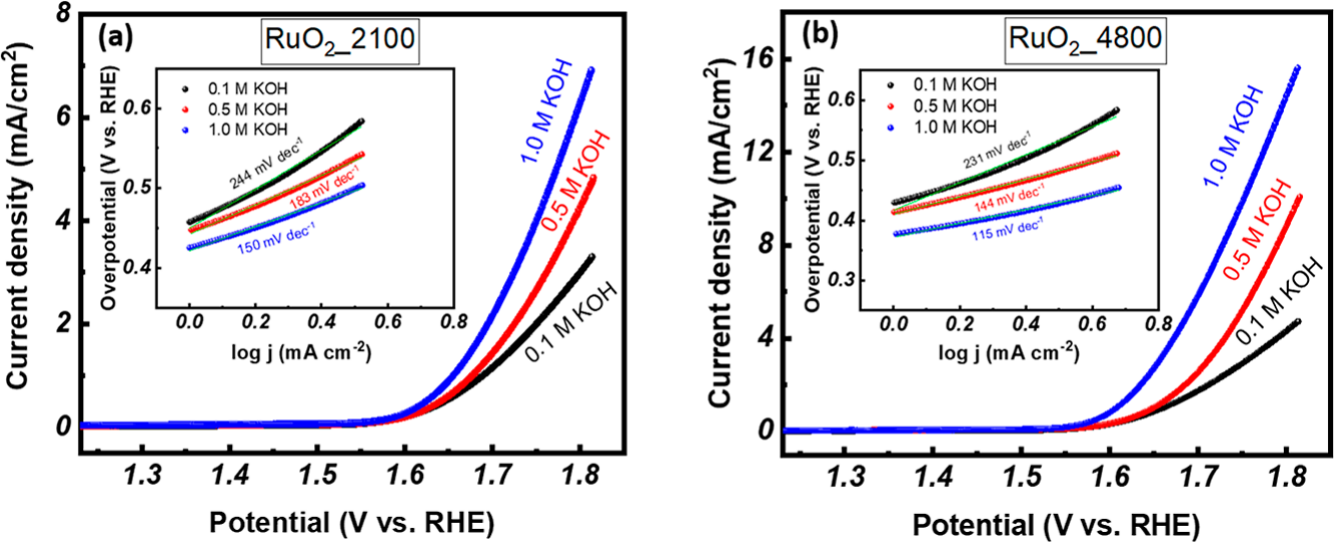
LSV curves for (a) RuO_2__2100 and (b) RuO_2__4800 samples, recorded in different concentrations of KOH electrolyte. Inset of Figure 7a,b shows the Tafel slope of RuO_2_ _2100 and RuO_2__4800 samples, respectively.

#### 3.2.4 Stability Tests Using Chronoamperometry and Pre- and Post-linear Sweep Voltammetry

Chronoamperometry (CA) test results for the RuO_2__2100 and RuO_2__4800 samples are shown in Figure 8a. The RuO_2__4800 sample exhibited remarkable stability, retaining 92% of its initial current density (applying various potentials such as 1.81, 1.76, 1.71, and 1.66 V vs RHE), while the RuO_2__2100 sample retained a current density of 83% of its initial value at over 2 h of testing. The CC test was also conducted in a fixed potential (1.81 V vs RHE) for 12 h (as shown in Figure S8), and the current density retention was 89% for the RuO_2_-4800 thin film, which is much higher than that of RuO_2_-2100 (51%). The electrochemical stability of each sample was also evaluated by recording LSV curves before and after CA tests. The LSV results are presented in Figure 8b. The RuO_2__4800 sample has better electrocatalytic activities than the RuO_2__2100 sample, as reflected in its nearly 9% less overpotential and almost 15% higher current density with respect to the RuO_2__2100 sample at 100 μA/cm^2^. The minor shift in the LSV curves of the RuO_2__4800 sample in comparison to that for the RuO_2__2100 sample (10 mV changes in the overpotential of the RuO_2__4800 sample versus 19 mV change in the overpotential for the RuO_2__2100 sample) can also be taken to suggest the stability of these films, making them viable candidates for efficient and stable OER electrocatalysts. To further assess the samples’ stability, corrosion tests were performed, and the results are presented in Figures S9a,b, and in Table S2. A comprehensive description of the experimental methodology and an in-depth discussion of the findings are provided in the Supporting Information.

**Figure 8:**
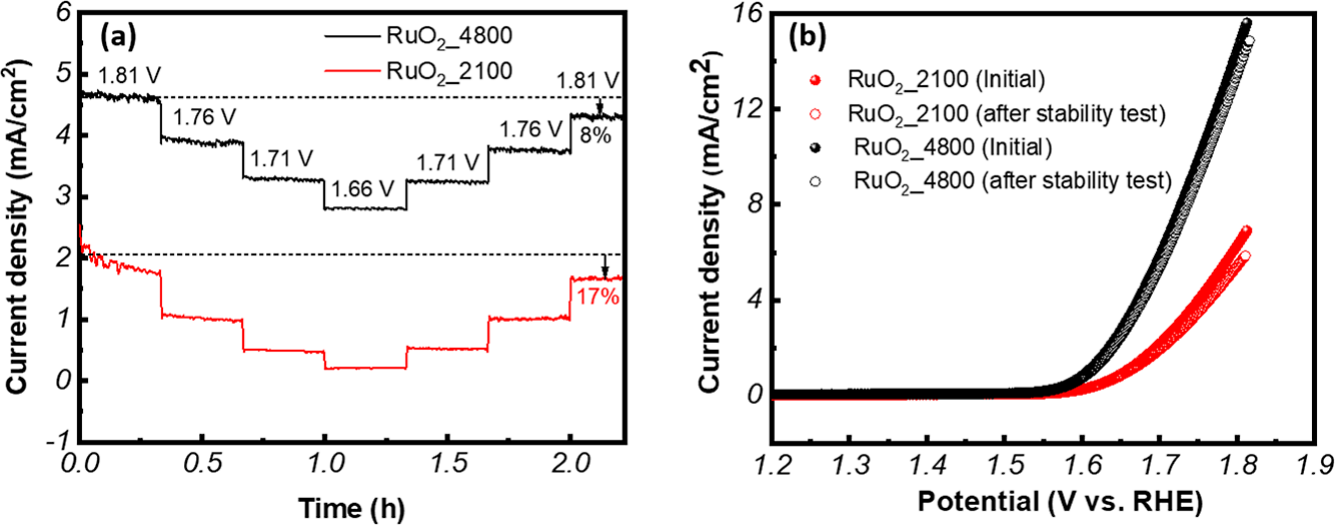
Chronoamperometric test results of RuO_2__2100 and RuO_2__4800 samples (a) applying different potential steps (0.85, 0.80, 0.75, and 0.70 V), and (b)­L SV curves of RuO_2__2100 and RuO_2__4800 samples in the initial stage and after the stability test.

Figure 9 displays resistivity as a function of temperature (300–570 K) for both RuO_2_ films made using 2100 (40 nm) and 4800 (87 nm), revealing a linear increase for both samples, indicative of metallic behavior. The temperature coefficient of resistivity (TCR = 1/(ρ_300_) * Δρ/Δ*T*, where ρ_300_ is resistivity at 300 K, Δρ is difference in resistivity, and Δ*T* is difference in temperature), which measures how much a material’s electrical resistivity changes with temperature, is higher for the thicker film (9.9 × 10^–4^ K^–1^) than for the thinner film (6.1 × 10^–4^ K^–1^).^67,68^

**Figure 9:**
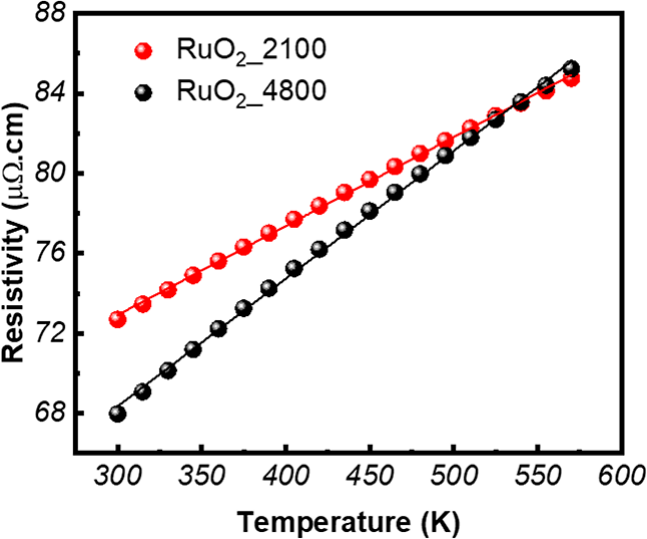
Four-probe resistivity as a function of temperature for RuO_2__2100 and RuO_2__4800 samples.

The sheet resistance (*R*_S_), electrochemical charge transfer resistance (*R*_CT_), and total mass for both sets of RuO_2_ films are shown in the bar charts in Figure S10. According to Figure S10, the RuO_2__2100 sample, which has 84% higher electrical resistance and 116% lower total mass, exhibits significantly higher (62%) charge transfer resistance compared to that of the RuO_2__4800 sample. Thus, these data seem to signify the effect of film’s thickness (or its body mass) and the absolute values of the electrical resistance, not the resistivity, on the electrocatalytic properties of RuO_2_ films in terms of LSV current density, overpotential, and EIS charge transfer resistance.

## 4 Conclusions

In this study, high-quality RuO_2_ thin films on sapphire substrates were successfully grown by using a PLD technique, and their electrochemical properties were reported. Among the available methodologies for synthesizing epitaxial thin films, PLD is particularly advantageous due to its low cost, simple operation, and high controllability of deposition parameters, yielding RuO_2_ epitaxial thin films with high precision in film stoichiometry, thickness, and crystallinity. Unlike chemical synthesis methods, PLD does not require hazardous gases or binder agents to attach electrode materials to the substrate, reducing contamination and preserving intrinsic material properties. Electrochemical evaluation of the RuO_2_ films for the OER revealed a realization of an onset potential as low as 1.51 V vs RHE at 100 μA/cm^2^, and a Tafel slope as low as 115 mV dec^–1^. The RuO_2__4800 films also exhibited higher specific capacitance, lower charge-transfer resistance, and greater stability over a range of potentials compared to the RuO_2__2100 thin films. These findings highlight the versatility of PLD and sapphire substrates in obtaining highly performing electrocatalytic RuO_2_ films, which are typically achieved by using more expensive thin-film techniques and substrates.

## Supplementary Material



## Notes

Current density as a function of the number pulses and thickness of RuO_2_ thin films; schematic of a pulsed laser deposition process; anodic and cathodic peak current density as a function of scan rate and the square root of scan rates for the RuO_2__2100 sample in (a) 0.5 M KOH and (b) 1.0 M KOH; anodic and cathodic peak current density as a function of scan rate and the square root of scan rates for the RuO_2__4800 sample in (c) 0.5 M KOH and (d) 1.0 M KOH; log­(*i*_p_) vs log­(*v*) plots for the RuO_2__2100 sample in (a) 0.5 M KOH and (b) 1.0 M KOH; log­(*i*_p_) vs log­(*v*) plots for the RuO_2__4800 sample in (c) 0.5 M KOH and (d) 1.0 M KOH; cyclic voltammetry curves at various scan rates for the RuO_2__2100 sample in (a) 0.1 M KOH, (b) 0.5 M KOH, and (c) 1.0 M KOH; cyclic voltammetry curves at various scan rates for the RuO_2__4800 sample in (d) 0.1 M KOH, (e) 0.5 M KOH, and (f) 1.0 M KOH; electrical double layer capacitance obtained from the slope of the plotting of current vs scan rate for (a) RuO_2__2100 sample and (b) RuO_2__4800 sample; specific capacitance obtained from integration of the electrostatic area of the CV curve for (c) RuO_2__2100 sample and (d) RuO_2__4800 sample; Bode plots for RuO_2_ _2100 and RuO_2_ _4800 samples under different concentrations of KOH electrolyte; all samples exhibit a clear phase minimum in the midfrequency region, indicating capacitive behavior, while the high-frequency region reflects the resistive contributions; the fitted curves show good agreement with the experimental data, confirming the reliability of the equivalent circuit model used to interpret charge transfer and double-layer properties; chronoamperometric test results for the RuO_2__2100 and RuO_2__4800 samples, recorded at an applied potential of 1.85 V vs RHE; corrosion test of (a) RuO_2__2100 and (b) RuO_2__4800 samples in 0.1, 0.5, and 1.0 M KOH solutions; the summary of corrosion current and potential of thin films in different concentration of electrolytes; and plotting of resistance/charge transfer resistance and total mass for the RuO_2__2100 and RuO_2__4800 samples (PDF)
