# Supplementary material for: Structural and Electrocatalytic Studies of Pulsed Laser Deposited Epitaxial RuO2 Thin Films
Source: ACS Appl Energy Mater. 2026 Jan 2;9(2):1080–91. doi: 10.1021/acsaem.5c03420 (PMC12848853; doi:10.1021/acsaem.5c03420)
Supplement: Supplementary file 1 [file ae5c03420_si_001.pdf]

Supporting information

Structural and Electrocatalytic Studies of Pulsed Laser Deposited Epitaxial RuO_2 Thin Films

Ghanashyam Gyawali[1], Mengxin Liu[1], Ikenna Chris-Okoro[1], Sheilah Cherono[1], Wisdom Akande[1], Brianna Barbee[1], Swapnil Nalawade[2], Jonathan Roop[1], Salil Pai[1], Shobha Mantripragada[2], Veluchamy P[1], Bishnu Prasad Bastakoti[3], Shyam Aravamudhan[2], Valentin Craciun[4,5], Maria Diana Mihai[6,7], Decebal Iancu[6], and Dhananjay Kumar[1*]

[1]Department of Mechanical Engineering, North Carolina A&T State University, Greensboro, NC 27411, USA

[2]Joint School of Nanoscience and Nanoengineering, North Carolina A&T State University, Greensboro, NC 27401, USA.

[3]Department of Chemistry, North Carolina A&T State University, Greensboro, NC 27411, USA.

[4]National Institute for Laser, Plasma and Radiation Physics, Romania 060042, Magurele, Romania

[5]Extreme Light Infrastructure for Nuclear Physics, HH-IFIN, Magurele, Romania

[6]Horia Hulubei National Institute for Physics and Nuclear Engineering, Măgurele, IF, 077125, Romania

[7]Faculty of Applied Sciences, National University of Science and Technology Politehnica Bucharest, 060042 Bucharest, Romania

*Correspondence: dkumar@ncat.edu

Contents

1. Figure S1. Figure S1. Current density as a function of RuO_2 thin-film thickness and number of laser pulses. The black circles represent the current density plotted as a function of film thickness, while the red triangles denote the current density as a function of the number of laser pulses.
2. Figure S2. Schematic of a pulsed laser deposition process.
3. Figure S3. Anodic and cathodic peak current density as a function of scan rate and the square root of scan rates for the RuO_2_2100 sample in (a) 0.5 M KOH and (b) 1.0 M KOH. Anodic and cathodic peak current density as a function of scan rate and the square root of scan rates for the RuO_2_4800 sample in (c) 0.5 M KOH and (d) 1.0 M KOH.
4. Figure S4. log(i_p) *vs.* log(v) plots for the RuO_2_2100 sample in (a) 0.5 M KOH and (b) 1.0 M KOH; log(i_p) *vs.* log(v) plots for the RuO_2_4800 sample in (c) 0.5 M KOH and (d) 1.0 M KOH.
5. Figure S5. Cyclic voltammetry curves at various scan rates for the RuO_2_2100 sample in (a) 0.1 M KOH, (b) 0.5 M KOH, and (c) 1.0 M KOH; cyclic voltammetry curves at various scan rates for the RuO_2_4800 sample in (d) 0.1 M KOH, (e) 0.5 M KOH, and (f) 1.0 M KOH.
6. Figure S6. Electrical double layer capacitance obtained from the slope of the plotting of current vs. scan rate for (a) RuO_2_2100 sample and (b) RuO_2_4800 sample; specific capacitance obtained from integration of the electrostatic area of the CV curve for (c) RuO_2_2100 sample and (d) RuO_2_4800 sample.
7. Figure S7. Bode plots for RuO_2 _2100 and RuO_2 _4800 samples under different concentrations of KOH electrolyte. All samples exhibit a clear phase minimum in the mid-frequency region, indicating capacitive behavior, while the high-frequency region reflects the resistive contributions. The fitted curves show good agreement with the experimental data, confirming the reliability of the equivalent circuit model used to interpret charge transfer and double-layer properties.
8. Figure S8. Chronoamperometric test results for the RuO_2_2100 and RuO_2_4800 samples, recorded at an applied potential of 1.85 V vs. RHE.
9. Figure S9. Corrosion test of (a) RuO_2_2100 and (b) RuO_2_4800 samples in 0.1 M, 0.5 M, and 1.0 M KOH solutions.

10. Table S1. The summary of corrosion current and potential of thin films in different concentration of electrolytes.

Figure S10. Plotting of resistance/charge transfer resistance and total mass for the RuO_2_2100 and RuO_2_4800 samples.

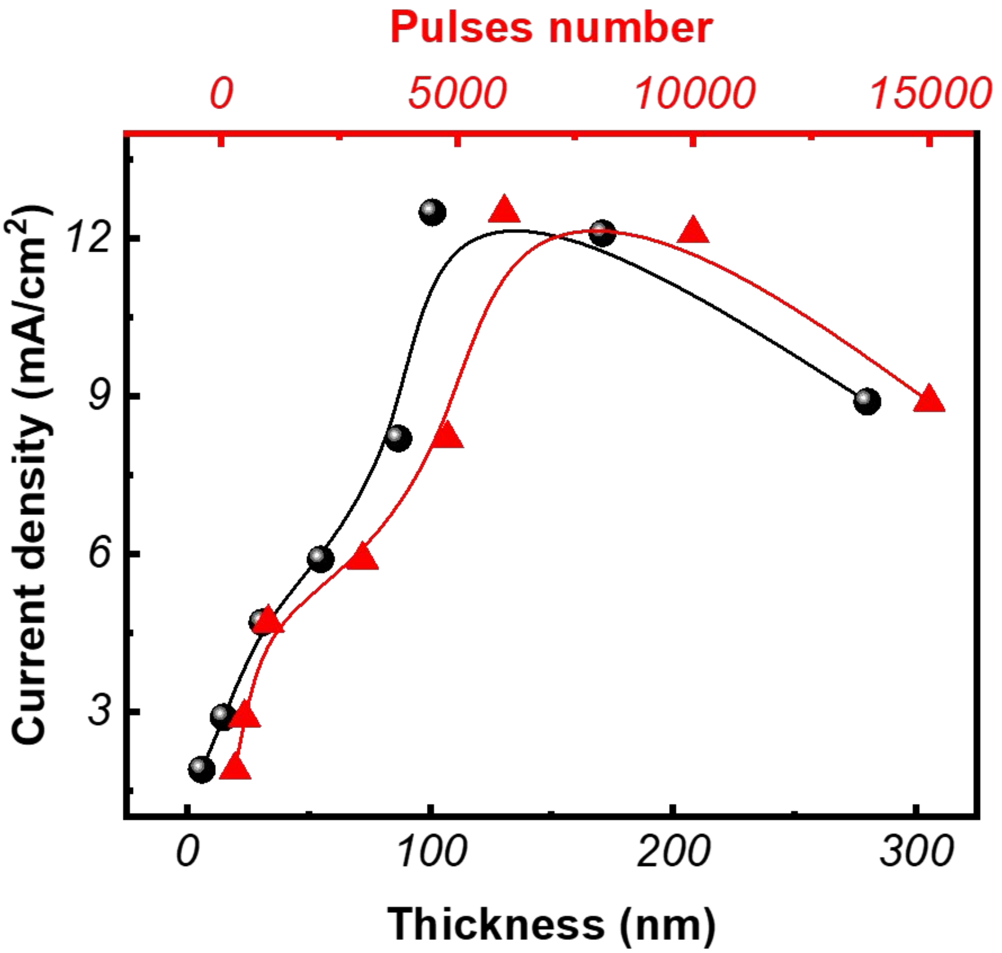

Figure S1. Current density as a function of RuO_2 thin-film thickness and number of laser pulses. The black circles (●) represent the current density plotted as a function of film thickness, while the red triangles (▲) denote the current density as a function of the number of laser pulses.

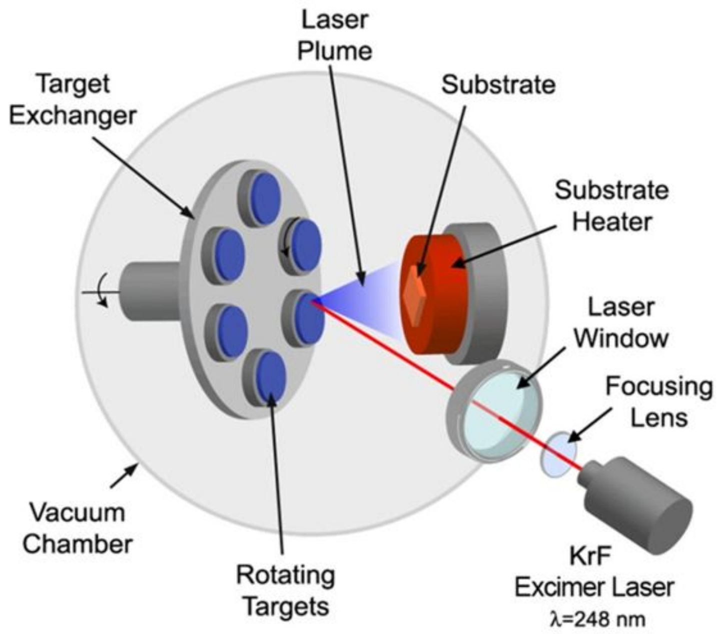

Figure S2. Schematic of a pulsed laser deposition process.

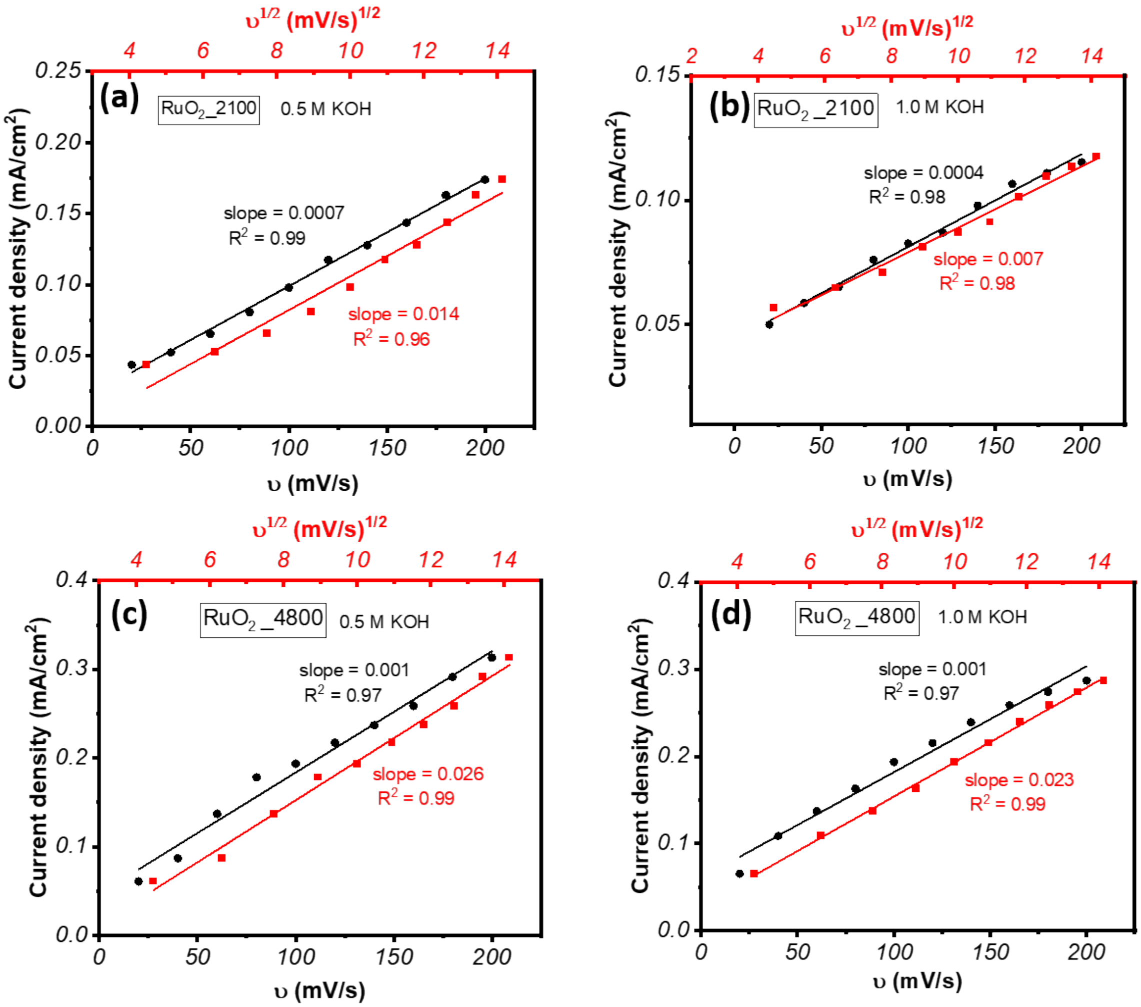

Figure S3. Anodic and cathodic peak current density as a function of scan rate and the square root of scan rates for the RuO_2_2100 sample in (a) 0.5 M KOH and (b) 1.0 M KOH. Anodic and cathodic peak current density as a function of scan rate and the square root of scan rates for the RuO_2_4800 sample in (c) 0.5 M KOH and (d) 1.0 M KOH.

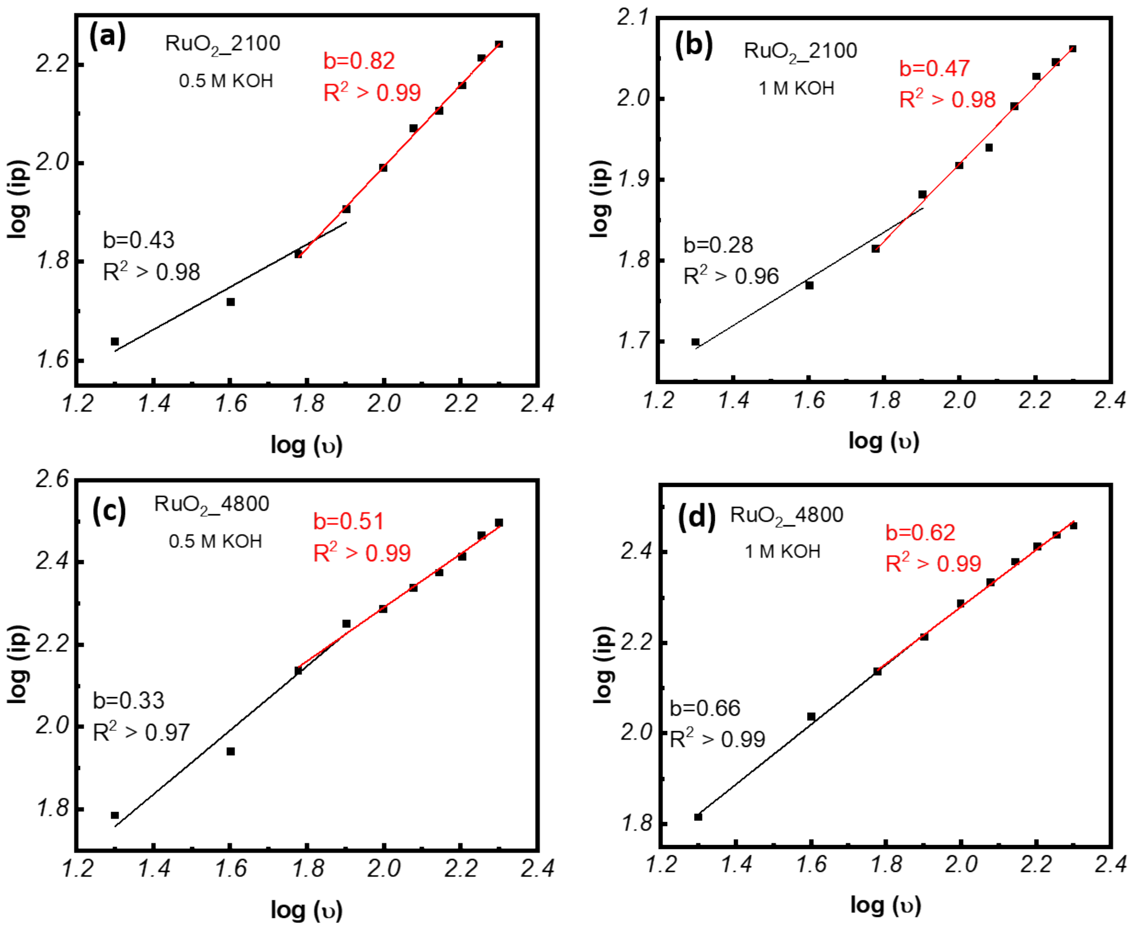

Figure S4. log(i_p) *vs.* log(v) plots for the RuO_2_2100 sample in (a) 0.5 M KOH and (b) 1.0 M KOH; log(i_p) *vs.* log(v) plots for the RuO_2_4800 sample in (c) 0.5 M KOH and (d) 1.0 M KOH.

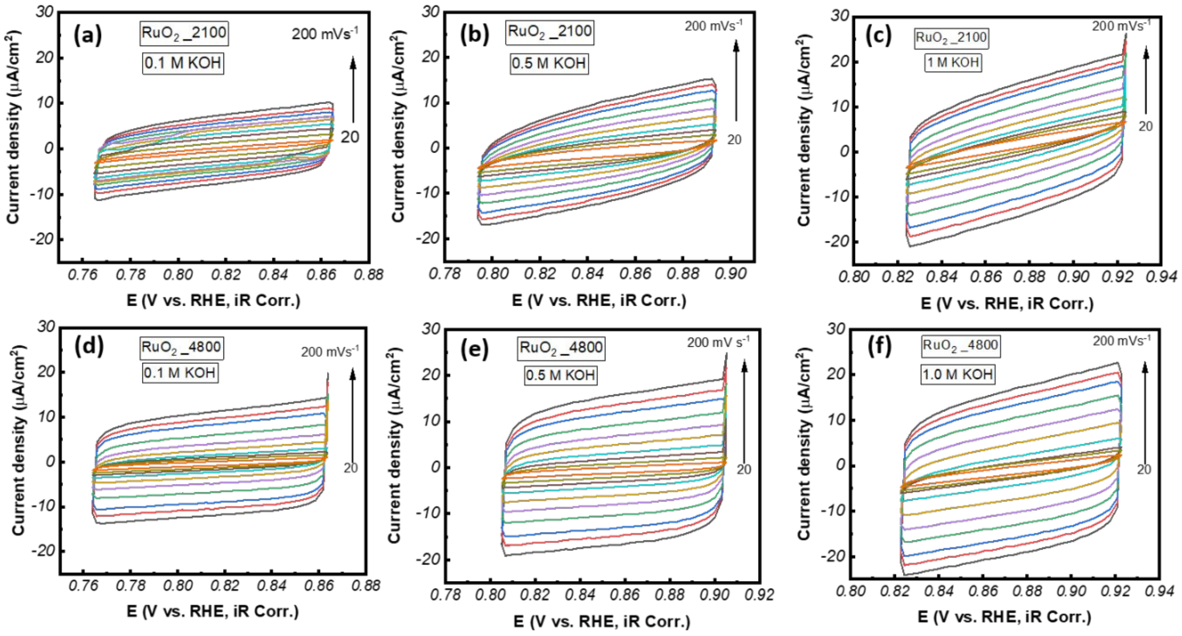

Figure S5. Cyclic voltammetry curves at various scan rates for the RuO_2_2100 sample in (a) 0.1 M KOH, (b) 0.5 M KOH, and (c) 1.0 M KOH; cyclic voltammetry curves at various scan rates for the RuO_2_4800 sample in (d) 0.1 M KOH, (e) 0.5 M KOH, and (f) 1.0 M KOH.

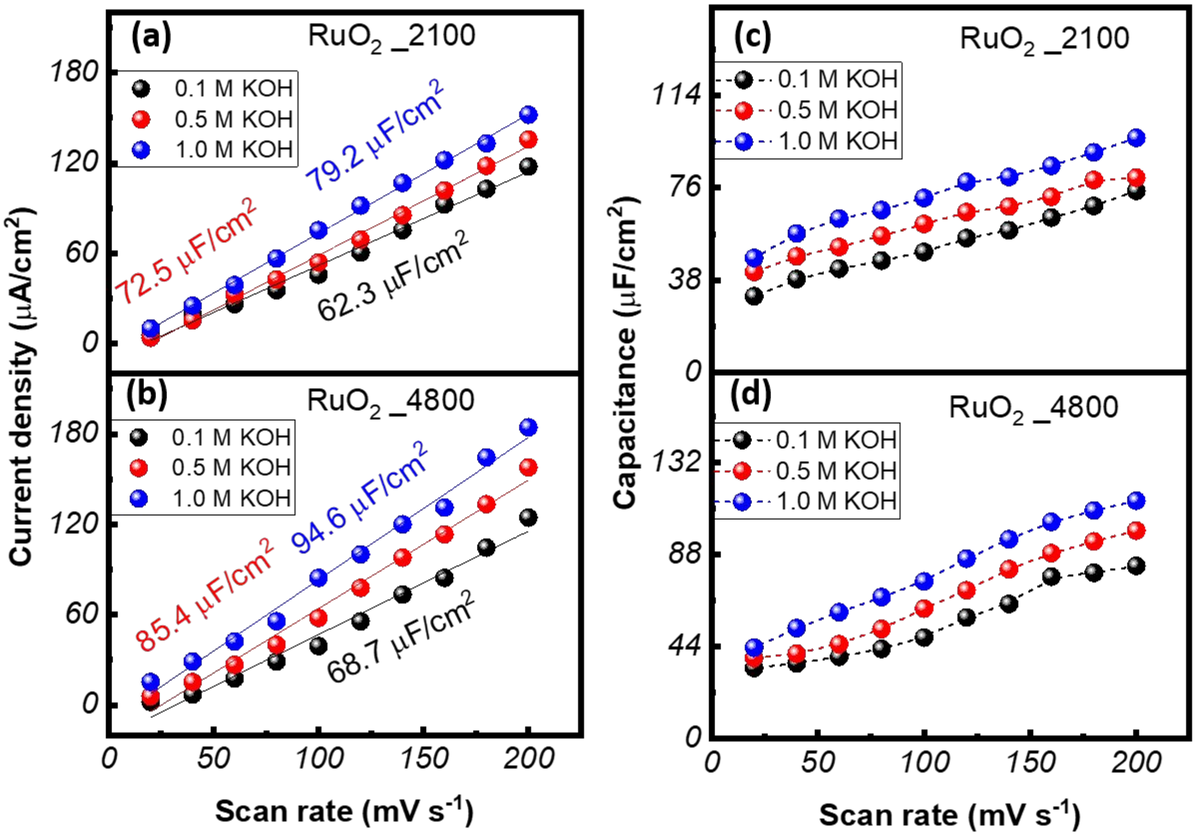

Figure S6. Electrical double layer capacitance obtained from the slope of the plotting of current vs. scan rate for (a) RuO_2_2100 sample and (b) RuO_2_4800 sample; specific capacitance obtained from integration of the electrostatic area of the CV curve for (c) RuO_2_2100 sample and (d) RuO_2_4800 sample.

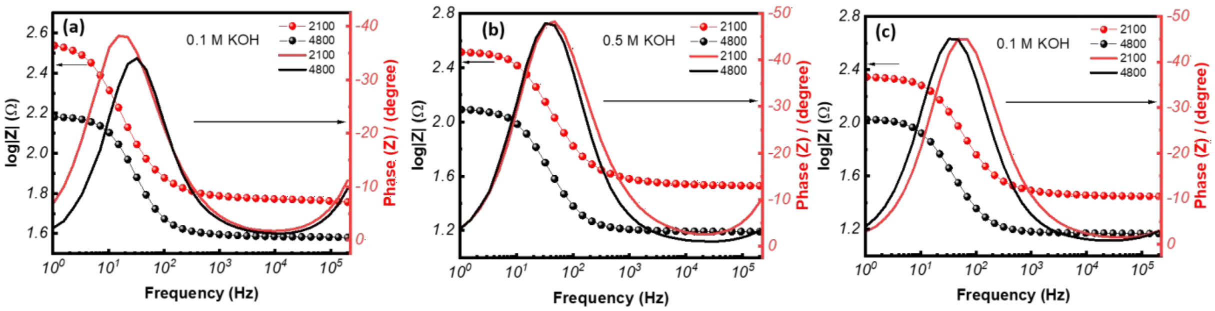

Figure S7. Bode plots for RuO_2 _2100 and RuO_2 _4800 samples under different concentrations of KOH electrolyte. All samples exhibit a clear phase minimum in the mid-frequency region, indicating capacitive behavior, while the high-frequency region reflects the resistive contributions. The fitted curves show good agreement with the experimental data, confirming the reliability of the equivalent circuit model used to interpret charge transfer and double-layer properties.

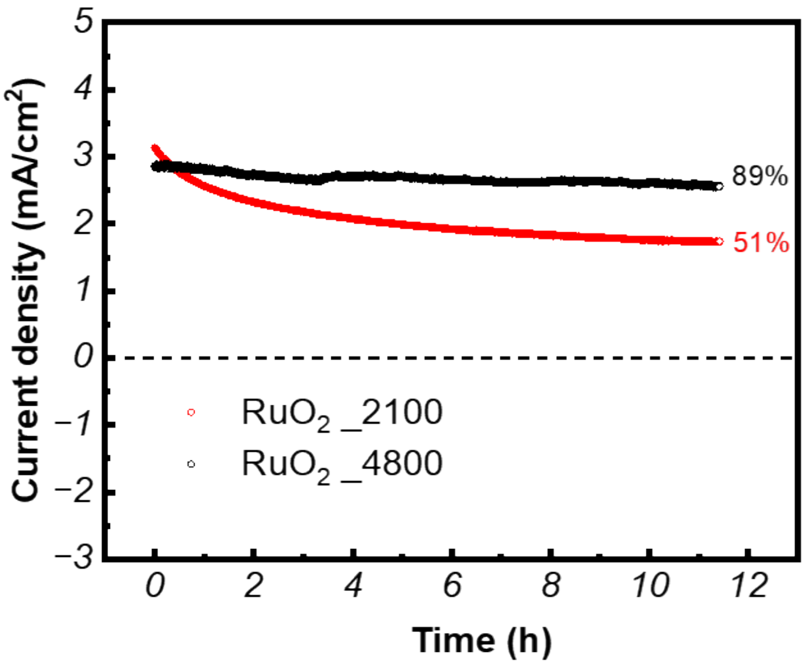

Figure S8. Chronoamperometric test results for the RuO_2_2100 and RuO_2_4800 samples, recorded at an applied potential of 1.85 V vs. RHE.

Corrosion test: The corrosion resistance of RuO_2 thin films was systematically investigated in 0.1 M, 0.5 M, and 1.0 M KOH electrolytes through Tafel polarization analysis. The corrosion potential (V) and corrosion current (log |i|) were extracted to assess the electrochemical stability of the films under alkaline conditions. As summarized in Table S1, both RuO_2-2100 and RuO_2-4800 thin films exhibit a trend of increasingly negative potential and elevated current values with increasing KOH concentration, indicating a higher corrosion rate in more concentrated electrolytes. Specifically, for RuO_2-2100, the potential shifted from -0.16 V in 0.1 M KOH to -0.20 V in 1.0 M KOH, while log |i| increased from 3.10 mA to 3.32 mA. In comparison, the RuO_2-4800 film demonstrated superior corrosion resistance, with less negative potential values (-0.11 V to -0.18 V) and lower log |i| values (3.02 mA to 3.31 mA) across the same concentration range. These results highlight that the increased pulse number during film deposition leads to improved structural integrity and passivation, which suppresses electrochemical degradation under alkaline conditions. Therefore, RuO_2-4800 exhibits enhanced long-term stability and is a more promising candidate for oxygen evolution reaction (OER) applications in alkaline media. The following equation describes the mechanism of the cathodic reaction.

$$O_2 + 2H_2O + 4e^- \rightarrow 4\ OH^-$$

The primary anodic reaction at the RuO_2 thin film electrode surface can be represented by the following reaction:

$$RuO_2 + 4OH^- \rightarrow RuO_4^{2-} + 2H_2O + 4e^-$$

This reaction highlights the oxidative dissolution of ruthenium species in alkaline media. The underlying Al_2O_3 substrate, being chemically inert and highly stable, provides structural support and does not participate in the electrochemical process. The increase in corrosion current with higher KOH concentration may also be attributed to enhanced accessibility of OH^- ions to the active sites on the RuO_2 film, promoting surface oxidation. These findings emphasize the need for careful control of electrolyte concentration in long-term electrochemical applications, as excessive alkalinity can compromise the durability of Ru-based electrode materials.

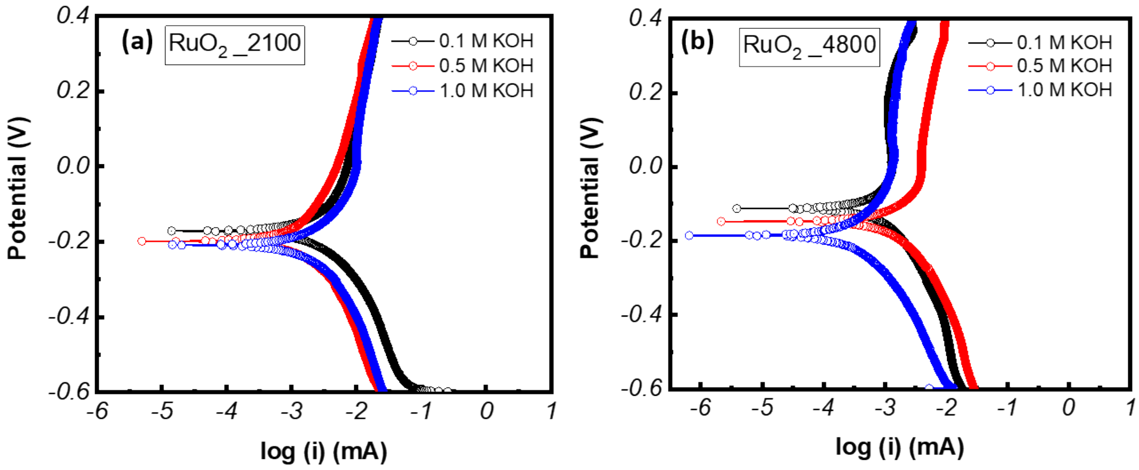

Figure S9. Corrosion test of (a) RuO_2_2100 and (b) RuO_2_4800 samples in 0.1 M, 0.5 M, and 1.0 M KOH solutions.

Table S1. Price of commonly used ceramic substrates for thin film electrocatalysts.

Substrate	Orientation	Dimension (mm)	Price ($)/unit
TiO_2	(100)	10 × 10 × 0.5	218
	(110)	10 × 10 × 0.5	187
	(101)	10 × 10 × 0.5	445
Al_2O_3	(0001)	10 × 10 × 0.5	29
$SrTiO_3$	(100)	10 × 10 × 0.5	63
	(110)	10 × 10 × 0.5	94
MgO	(100)	10 × 10 × 0.5	36
	(110)	10 × 10 × 0.5	43

*Source: www.crystalsubstrates.com

Table S2. The summary of corrosion current and potential of thin films in different concentration of electrolytes.

Sample	Electrolyte concentration (KOH)	Corrosion current log |i| (mA)	Corrosion Potential (V vs. Ag/AgCl)
RuO_2 _2100	0.1 M	3.10	-0.16
	0.5 M	3.23	-0.19
	1.0 M	3.33	-0.20
RuO_2 _4800	0.1 M	3.02	-0.11
	0.5 M	3.13	-0.14
	1.0 M	3.31	-0.18

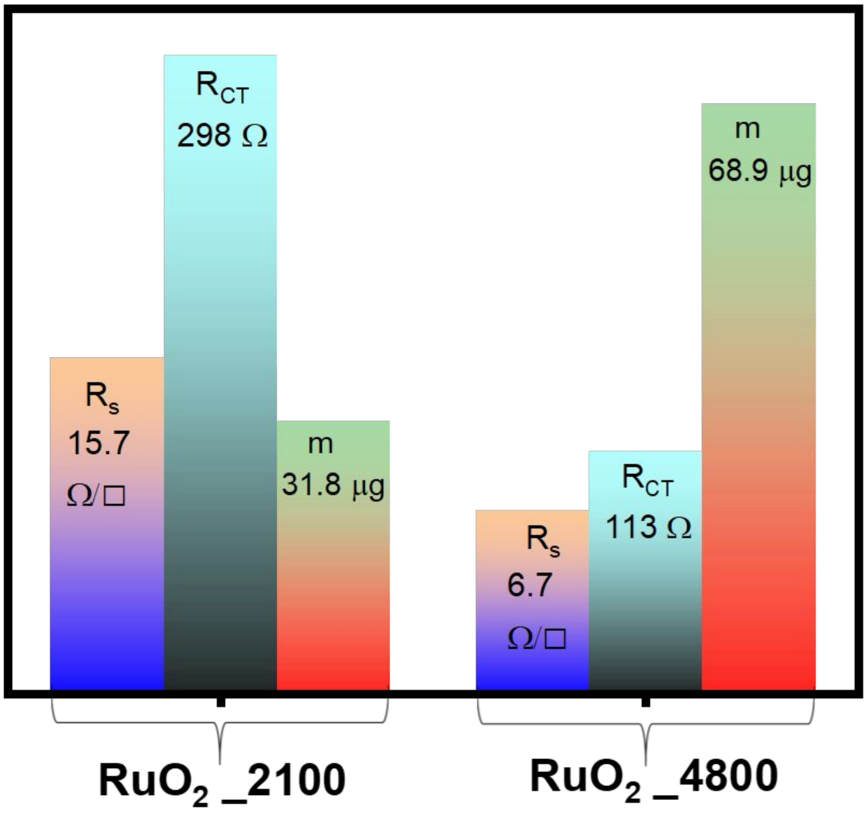

Figure S10. Plotting of resistance/charge transfer resistance and total mass for the RuO_2_2100 and RuO_2_4800 samples.
